# Uniaxial Cyclic Stretching Promotes Chromatin Accessibility of Gene Loci Associated With Mesenchymal Stem Cells Morphogenesis and Osteogenesis

**DOI:** 10.3389/fcell.2021.664545

**Published:** 2021-07-07

**Authors:** Duo Zhang, Ran Zhang, Xiaoyuan Song, Karen Chang Yan, Haiyi Liang

**Affiliations:** ^1^CAS Key Laboratory of Mechanical Behavior and Design of Materials, Department of Modern Mechanics, University of Science and Technology of China, Hefei, China; ^2^Hefei National Laboratory for Physical Sciences at the Microscale, CAS Key Laboratory of Brain Function and Disease, Division of Life Sciences and Medicine, School of Life Sciences, University of Science and Technology of China, Hefei, China; ^3^Mechanical Engineering and Biomedical Engineering, The College of New Jersey, Ewing Township, NJ, United States

**Keywords:** mesenchymal stem cells, uniaxial cyclic stretching, differentiation, chromatin accessibility, mechanotransduction, mechanobiology, tissue engineering

## Abstract

It has been previously demonstrated that uniaxial cyclic stretching (UCS) induces differentiation of mesenchymal stem cells (MSCs) into osteoblasts *in vitro*. It is also known that interactions between cells and external forces occur at various aspects including cell–matrix, cytoskeleton, nucleus membrane, and chromatin. However, changes in chromatin landscape during this process are still not clear. The present study was aimed to determine changes of chromatin accessibility under cyclic stretch. The influence of cyclic stretching on the morphology, proliferation, and differentiation of hMSCs was characterized. Changes of open chromatin sites were determined by assay for transposase accessible chromatin with high-throughput sequencing (ATAC-seq). Our results showed that UCS induced cell reorientation and actin stress fibers realignment, and in turn caused nuclear reorientation and deformation. Compared with unstrained group, the expression of osteogenic and chondrogenic marker genes were the highest in group of 1 Hz + 8% strain; this condition also led to lower cell proliferation rate. Furthermore, there were 2022 gene loci with upregulated chromatin accessibility in 1 Hz + 8% groups based on the analysis of chromatin accessibility. These genes are associated with regulation of cell morphogenesis, cell–substrate adhesion, and ossification. Signaling pathways involved in osteogenic differentiation were found in up-regulated GO biological processes. These findings demonstrated that UCS increased the openness of gene loci associated with regulation of cell morphogenesis and osteogenesis as well as the corresponding transcription activities. Moreover, the findings also connect the changes in chromatin accessibility with cell reorientation, nuclear reorientation, and deformation. Our study may provide reference for directed differentiation of stem cells induced by mechanical microenvironments.

## 1. Introduction

Mesenchymal stem cells (MSCs) have been widely studied for cell-based therapies and tissue regeneration as MSCs are multipotent cells that have the ability of differentiating into a various lineage of cells such as osteoblasts, chondrocytes, and adipocytes (Pittenger et al., 1999; Kolf et al., 2007; Uchibori et al., 2014; Marfia et al., 2015; Yorukoglu et al., 2017; Zwolanek et al., 2017). The lineage commitment of MSCs is influenced by various factors including biochemical and mechanical signals. Existing studies have identified a number of signaling pathways that regulate the linage commitment, such as transforming growth factor-beta (TGF-β)/bone morphogenic protein (BMP) signaling (Chen et al., 2012b; Wang et al., 2012; Wu et al., 2016), Wnt signaling (Day et al., 2005; Baron and Kneissel, 2013), RhoA signaling (McBeath et al., 2004), and mitogen-activated protein kinase (MAPK) signaling pathway (Jaiswal et al., 2000). Well-established culture media contain relevant biochemical cues to guide specific differentiation. For instance, insulin-like growth factor-I (IGF-I) and dexamethasone treatment increase the rate of bone matrix formation and induce osteoblasts differentiation of human bone marrow stromal cells (Hock et al., 1988; Cheng et al., 1994). Moreover, it is also well-known that MSCs sense and interact with surrounding mechanical environment constantly. Extensive research has shown that mechanical factors play important and integral roles in MSCs differentiation. These factors include substrate stiffness and pattern (Engler et al., 2006; Li et al., 2006; Dalby et al., 2007; Oh et al., 2009; Fu et al., 2010; Kilian et al., 2010; Trappmann et al., 2012), 3D extracellular matrix (ECM) structure and stiffness (Huebsch et al., 2010; Pek et al., 2010; Khetan et al., 2013; Chaudhuri et al., 2016), and external mechanical forces (Jagodzinski et al., 2004; Qi et al., 2008; Kang et al., 2012; Carroll et al., 2017).

Mechanical interactions between cells and external forces occur at various levels including cell–matrix, cytoskeleton, nucleus membrane, and chromatin. Existing works have demonstrated that these interactions lead to formation of focal adhesions (Na et al., 2008), cytoskeleton reorganization (Geiger et al., 2006), and deformation of nucleus (Heo et al., 2016a). Moreover, research efforts have also shed lights on biochemical processes triggered/activated by external forces due to these mechanical interactions and corresponding mechanisms (Kaunas et al., 2005; Torsoni et al., 2005; Dupont et al., 2011; Xu et al., 2012). In recent years, an emerging understanding is that mechanical signals that transmit to the nucleus can activate different gene expression programs and regulate transcription factors (Arnsdorf et al., 2009; Iyer et al., 2012; Heo et al., 2016b; Tajik et al., 2016; Athirasala et al., 2017; Miroshnikova et al., 2017; Uhler and Shivashankar, 2017). Several mechanisms have been proposed for how the cell nucleus can directly response to mechanical forces. One of these mechanisms is that external forces can induce chromatin stretching, altering polymerase and transcription factor accessibility and activity (Kirby and Lammerding, 2018). However, little is known about the transcriptional regulatory network in this process due to the inherent complexity. For instance, whether chromatin stretching lead to similar accessibility changes for genomic loci in the same region? What are specific genomic loci influenced by chromatin stretching in terms of the accessibility of the transcription sites?

To this end, the objective of this study was to examine changes in open chromatin sites through chromatin accessibility analysis for hMSCs subjected to uniaxial cyclic stretching (UCS). Specifically, we designed a mechanical device and applied force to the hMSCs through elastic substrate. We then characterized the influence of cyclic stretching on the morphology, proliferation, and differentiation of hMSCs in the absence of specific growth factors. Finally, changes of open chromatin sites were determined by assay for transposase accessible chromatin with high-throughput sequencing (ATAC-seq). The results of the present study demonstrated that UCS increases accessibility of the gene loci associated with hMSCs morphogenesis and osteogenesis. Furthermore, our findings also connect the changes in chromatin accessibility with cell reorientation, nuclear reorientation, and deformation.

## 2. Materials and Methods

### 2.1. Cells Culture and Y27632 Treatment

The hMSCs derived from umbilical cord were purchased from Nuwacell Co., Ltd. (Hefei, China) as stem cells product. Following the company's protocols, hMSCs were cultured in ncMission Basal Medium (RP02010-01, Nuwacell) supplemented with ncMission 25 × Supplement (RP020210-02, Nuwacell). Culture dishes were kept at 37°C in a humidified incubator with 5% CO_2_. Complete medium was changed twice per week. When hMSCs reached 80% confluency, cells were released with 0.25% trypsin (SH30042.01, HyClone) and plated at a density of 5 × 10^3^/cm^2^. The hMSCs at passages 6–7 were used in our experiments. To verify the effect of GTPase activity on cell morphology and nuclear deformation in this UCS platform, hMSCs were incubated in culture media with 10 μM Y27632 (SC0326, Beyotime) to inhibit Rho-kinase activity. The cells were pretreated 30 min before UCS, and Y27632 was present throughout the experiment.

### 2.2. Cells Seeding and Mechanical System Setup

**Figure 1:**
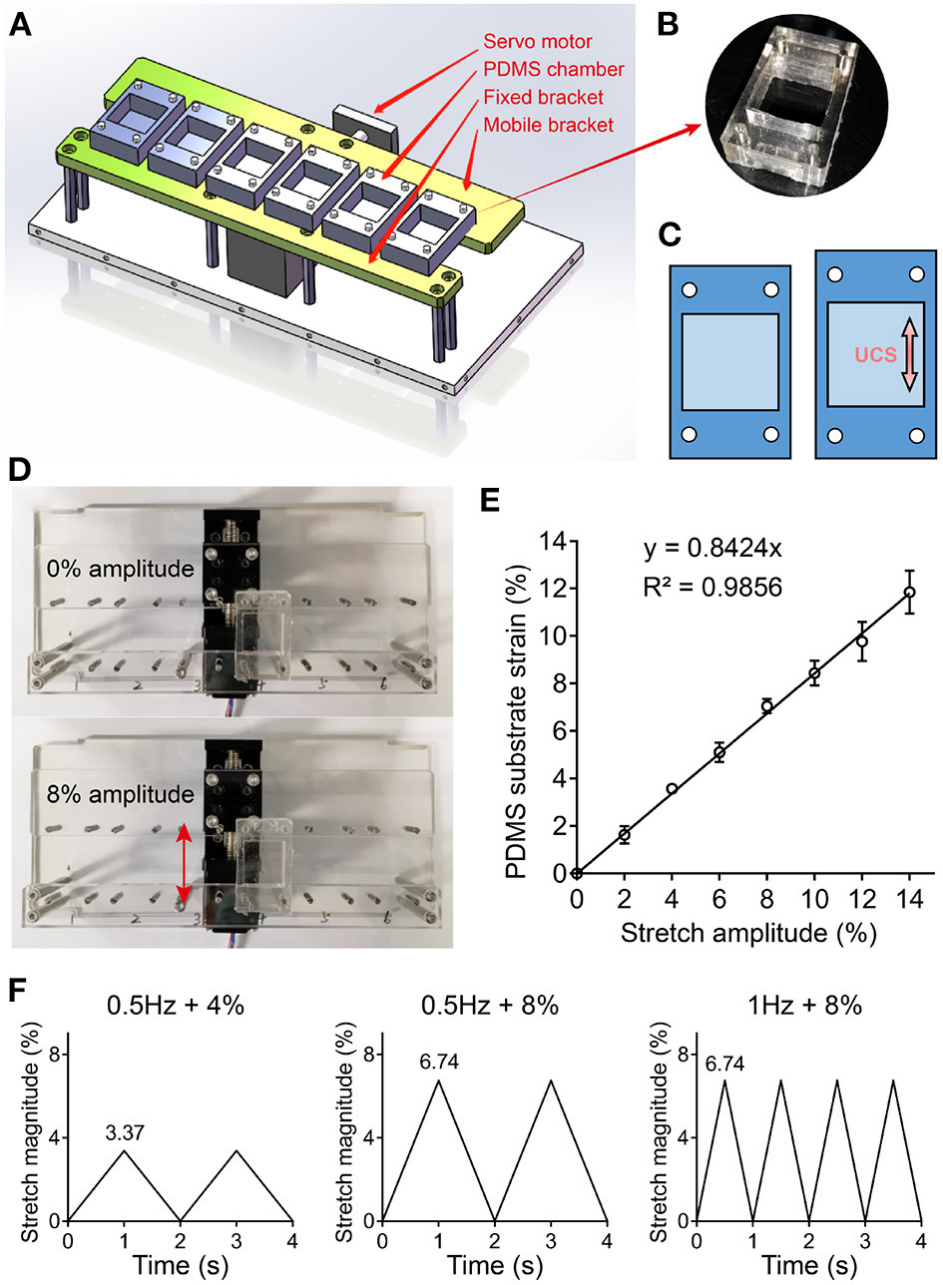
**(A)** 3D drawing of an uniaxial cyclic stretchable homemade device with a servo motor, fixed and mobile bracket, and six elastic polydimethylsiloxane (PDMS) chambers. The device was driven by the servo motor that can load different frequencies and amplitudes of mechanical stimulation. **(B)** Photograph of PDMS chamber containing a 4 cm^2^ cell culture area in the center. **(C)** Schematic illustration of PDMS substrate deformation. **(D,E)** Tensile testing analyses were performed to correlate stretch amplitude with PDMS substrate strain. Representative images **(D)** and data **(E)** of PDMS substrate deformation under different stretch amplitude are shown. **(F)** Three kinds of cyclic stretch with triangular wave pattern are shown. The 4 and 8% amplitude were servo motor set value, and the actual strain of PDMS substrate were 3.37 and 6.74%, respectively.

UCS was applied to the hMSCs mono-layers using the uniaxial cyclic stretchable homemade device, which consists of controller, dynamic bracket, and elastic cell culture chambers made up of polydimethylsiloxane (PDMS) (Figures 1A,B). The device was driven by a servo motor that allows for mechanical stimulations with varying frequency and amplitude (Figure 1C). The PDMS (Sylgard 184, Dow Corning) chamber was formed by pouring the mixture of Part A and B (10:1 ratio) into acrylic molds and then cured at 100°C for 1 h. Prior to cell culture, the shaped chambers were sterilized using autoclave and coated with 1% gelatin (V900863; Sigma Aldrich). hMSCs were seeded on the PDMS chambers with a density of 1.5 × 10^4^/cm^2^. After 24 h of incubation to allow cells attachment and spreading, the medium was replaced with fresh medium to remove unadherent cells. Loading chambers onto holder, hMSCs were then subjected to UCS for 48 h. The unstrained hMSCs were cultured on the same chambers and maintained in the same incubator as control. Tensile testing analyses of PDMS chamber being strained were performed to correlate PDMS substrate deformation with stretch amplitude (Figures 1D,E). The actual strain of PDMS substrate was 84% of the servo motor setup (Figure 1E). The 4 and 8% amplitude mentioned later were servo motor set value, and the actual strain of PDMS substrate were 3.37 and 6.74%, respectively. Three cyclic stretch patterns in this study were all in the form of triangular wave pattern (Figure 1F).

### 2.3. Cells Morphology Assay

To compare the morphology and alignment of hMSCs between unstrained and three strained groups, we obtained microscopic images of hMSCs using a microscope (IX73; Olympus) in at least three randomly visual fields after UCS for 0 and 48 h. Statistical distribution of angles between the direction of cell spreading and UCS was analyzed by ImageJ software (National Institutes of Health, Bethesda, MD). The direction of the cell long axis indicates the direction of cell alignment. ImageJ software was used to measure the long axis of cells with clear contours. For a small number of cells with unclear contours, the orientation of the long axis was determined manually.

### 2.4. F-Actin Staining

To visualize the cytoskeleton structure of hMSCs, actin filaments were marked by fluorescent phalloidin. After mechanical loading, cells cultured in PDMS chambers were fixed in 4% paraformaldehyde solution for 10 min and rinsed with PBS. Then, hMSCs were permeabilized with 0.1% Triton X-100 for 5 min, and blocked with 1% bovine serum albumin at room temperature. Thereafter, cells were incubated in iFluor 647 phalloidin (MB5939, Meilunbio) working solution for 30 min to label actin and in Hoechst 33342 (14533; Sigma-Aldrich) working solution for 10 min to label the nuclei. After washing with PBS, the cytoskeleton was visualized by using 650 nm excitation and 670 nm emission under fluorescent microscope (IX73; Olympus) with DP80 camera.

### 2.5. F-Actin Mean Fluorescence Intensity and Nuclear Aspect Ratio Measurement

Fluorescence images were split into single channel images using ImageJ software. The gray value of each pixel represents the fluorescence intensity of the point. Mean fluorescence intensity (MFI), mean gray value, was calculated using integrated intensity per unit area. Nuclear aspect ratio (NAR) was calculated as the ratio of major axis to minor axis of the nucleus. The major and minor axes were obtained from the fitted ellipse of a nuclear contour using ImageJ software.

### 2.6. MTT Assay

MTT (3-(4,5-dimethylthiazol-2-yl)-2,5-diphenyltetrazolium bromide) staining assay was used to measure the relative mitochondrial activities of hMSCs under mechanical loading. hMSCs were seeded on PDMS chamber at a density of 1.0 × 10^4^/cm^2^ and attached substrate for 24 h. After cells were subjected to UCS for 48 h, culture medium was removed from the chambers, and the chambers were maintained in 0.5 mg/mL MTT solution (MA0198; Meilunbio) in incubator for 4 h to form purple formazan crystals. These crystals were incubated for another 4 h and dissolved in DMSO. The absorbance of each group was measured by enzyme-labeled instrument at 570 nm.

### 2.7. EdU Assay

Cell proliferation assay was performed using BeyoClick^*TM*^ EdU Cell Proliferation Kit with Alexa Fluor 647 (C0081S; Beyotime). hMSCs were seeded on PDMS chamber at a density of 1.0 × 10^4^/cm^2^ and attached substrate for 24 h. Subjected to UCS for 48 h, the cells were incubated with 10 μm EdU for 12 h at 37°C. Then, the hMSCs were fixed in 4% paraformaldehyde solution for 10 min and rinsed with PBS. After permeabilized with 0.1% Triton X-100 for 5 min and rinsed with PBS, the hMSCs were exposed to 500 μL click reaction cocktail for 30 min and then incubated with Hoechst 33342 (14533; Sigma-Aldrich) working solution for 10 min to stain the nuclei. Images were captured by using 650 nm excitation and 670 nm emission under fluorescent microscope (IX73, Olympus) with DP80 camera. The percentage of EdU-positive cells was defined as the proliferation rate.

### 2.8. TUNEL Assay

To measure the apoptosis of hMSCs under mechanical loading, TdT-UTP nick, end labeling (TUNEL) assay was performed using One Step TUNEL Apoptosis Assay Kit (C1086; Beyotime). hMSCs were seeded on PDMS chamber at a density of 1.0 × 10^4^/cm^2^ and attached substrate for 24 h. After mechanical loading, the cells cultured in PDMS chambers were fixed in 4% paraformaldehyde solution for 10 min and rinsed with PBS, permeabilized with 0.1% Triton X-100 for 5 min, and followed by TUNEL for 1 h at 37°C. The TUNEL-positive cells were imaged by using 488 nm excitation and 530 nm emission under fluorescent microscope (IX73; Olympus) with DP80 camera. The cells with green fluorescence were defined as apoptotic cells. To verify the validity of this experiment, the positive control of TUNEL assay in culture dish was treated with DNase I (50 U/μL) at 37°C for 30 min after fixed and permeabilized, and followed by TUNEL.

### 2.9. RNA Isolation and RT-qPCR

Total RNA of hMSCs from unstrained and UCS groups were extracted using TRIzol reagent (Invitrogen) according to the manufacturer protocol. The purity and concentration of the total RNA were determined by Nano-300 spectrophotometer with the 260/280 absorbance ratio. Reverse transcription was completed using HiScript II 1st Strand cDNA Synthesis Kit (R211, Vazyme) with 1 μg RNA according to the manufacturer protocol. Real-time qPCR reactions were performed in 10 μL volumes containing 5 μL AceQ qPCR SYBR Green Master Mix (Q111-02, Vazyme), 0.5 μM forward and reverse primers (General Bio), 50 ng cDNA, and RNase-free water. Reactions were carried out on the CFX connect real-time PCR detection system (Bio-Rad) with an amplification profile: 95°C for 5 min, 40 cycles of denaturation at 95°C for 30 s, annealing at 58°C for 30 s, and amplification at 72°C for 10 s. Quantitative expression of target mRNAs were determined by using gene-specific primer pairs:

*BMP2* (5′-ACTACCAGAAACGAGTGGGAA-3′, 5′-GCATCTGTTCTCGGAAAACCT-3′),

*RUNX2* (5′-TGGTTACTGTCATGGCGGGTA-3′, 5′-TCTCAGATCGTTGAACCTTGCTA-3′),

*ALPL* (5′-ACCACCACGAGAGTGAACCA-3′, 5'-CGTTGTCTGAGTACCAGTCCC-3'),

*SPP1* (5′-GAAGTTTCGCAGACCTGACAT-3′, 5′-GTATGCACCATTCAACTCCTCG-3′),

*BGLAP* (5′-CACTCCTCGCCCTATTGGC-3′, 5′-CCCTCCTGCTTGGACACAAAG-3′),

*COL10A1* (5′-CATAAAAGGCCCACTACCCAAC-3′, 5′-ACCTTGCTCTCCTCTTACTGC-3′),

*COL2A1* (5′-TGGACGCCATGAAGGTTTTCT-3′, 5′-TGGGAGCCAGATTGTCATCTC-3′),

*SOX9* (5′-AGCGAACGCACATCAAGAC-3′, 5′-CTGTAGGCGATCTGTTGGGG-3′),

*FABP4* (5′-ACTGGGCCAGGAATTTGACG-3′, 5′-CTCGTGGAAGTGACGCCTT-3′),

*ID2* (5′-AGTCCCGTGAGGTCCGTTAG-3′, 5′-AGTCGTTCATGTTGTATAGCAGG-3′),

*NCAM1* (5′-GGCATTTACAAGTGTGTGGTTAC-3′, 5′-TTGGCGCATTCTTGAACATGA-3′),

*ACTA2* (5′-GTGTTGCCCCTGAAGAGCAT-3′, 5′-GCTGGGACATTGAAAGTCTCA-3′),

*CNN1* (5′-GAACGTGGGAGTGAAGTACGC-3′, 5′-CAGCCCAATGATGTTCCGC-3′),

*TNC* (5′-AGGGCAAGTGCGTAAATGGAG-3′, 5′-TGGGCAGATTTCACGGCTG-3′),

*TNMD* (5′-CCATGCTGGATGAGAGAGGTT-3′, 5′-TTGGTAGCAGTATGGATATGGGT-3′),

*GAPDH* (5′-CATGTTCGTCATGGGTGTGAACCA-3′, 5′-ATGGCATGGACTGTGGTCATGAGT-3′).

All data were normalized to the reference gene *GAPDH*.

### 2.10. ATAC-seq Experiments

Tn5 transposase enzyme was received from Dr. Tengchuan Jin, University of Science and Technology of China. 10^5^ cells were lysed with cold resuspension buffer (10 mM Tris-HCl pH 7.4, 10 mM NaCl, and 3 mM MgCl_2_ in water) containing 0.1% NP40, 0.1% Tween-20, and 0.01% digitonin and centrifuged 500 g for 5 min at 4°C. The pellet was resuspended in 100 μL transposase mixture (50 μL 2 × TD buffer, 4 μL transposase (100 nM final), 33 μL PBS, 1 μL 1% digitonin, 1 μL 10% Tween-20, and 11 μL water). Transposition reactions were incubated at 37°C for 30 min in a thermomixer with shaking at 10^3^ r.p.m. After transposition, the mixture was purified with HiPure Gel Pure DNA mini kit (D2111; Magen). Sequencing libraries were prepared following the original ATAC-seq protocol (Buenrostro et al., 2013).

### 2.11. Statistical Analysis

For RT-qPCR, each experiment was performed with a minimum number of technical triplicates, and *n* indicates the number of replicates in each group. All RT-qPCR data were expressed as mean ± SD. Statistical analyses were performed using GraphPad Prism software. The unpaired *t*-tests were used to compare two groups, and multiple comparisons among the groups were performed using one-way ANOVA with Bonferroni's *post hoc* test. Statistical significance was accepted at a value of *P* value < 0.05. For ATAC-seq, Nextera adaptor sequences were trimmed from the reads by using cutadaptor v1.9.1. ATAC-seq paired-end reads were mapped to hg19 using Bowtie2 v2.2.5 with parameters –3 120. Samtools v1.7 was then used to remove duplicated reads and chromosome M. Peak calling was performed by Genrich v0.5 ATAC-seq mode. The intersect function of BedTools v2.25.0 was used to count the number of reads mapped to each peak. The count matrix was normalized by Reads Per Million (RPM) mapped reads. Pearson correlation coefficients between biological replicates were calculated based on the Log10 RPM matrix. The peak location in terms of genomic features was confirmed by ChIPseeker and TSS is defined from –3 to +3 kb. Gene Ontology (GO) enrichment was performed with clusterProfiler. *P* values were adjusted with “BH” method. GO categories with *q* < 0.05 were consider as significant. DiffBind was used to do principal components analysis and identify variable peaks between unstrained and 1 Hz + 8% group. The transcription factors motif enrichment analysis of peaks located in promoter regions was performed using HOMER with options: findmotifs.pl input.fa fasta output.

## 3. Results

### 3.1. UCS Induces Reorientation and Nuclear Deformation of hMSCs

**Figure 2:**
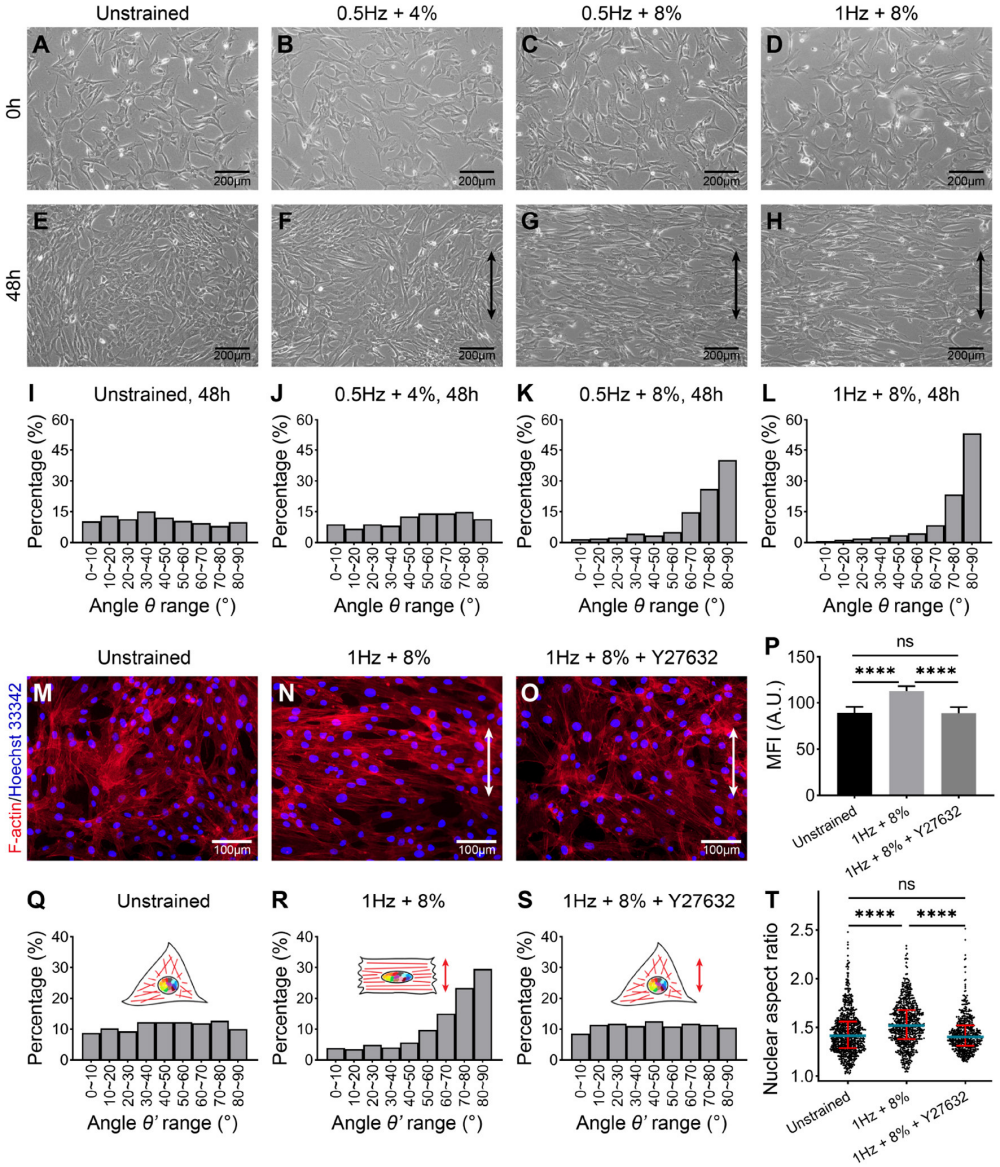
Phase-contrast images of hMSCs cultured on polydimethylsiloxane (PDMS) for 24 h before uniaxial cyclic stretching (UCS) **(A–D)** and subjected to UCS at different amplitude and frequency for 48 h **(E–H)**. Black double-headed arrows indicate the direction of stretching. **(I–L)** Distribution analysis of angle θ between the direction of cell spread (shown in **E–H**) and cyclic stretch. Every 10° is a range. Numbers of hMSCs measured orientation angle in each group **(I–L)** were 484, 434, 413, and 304, respectively. The cells from four biological replicates were counted together. **(M–O)** Representative fluorescence images of F-actin (red) and nuclei (blue) staining of hMSCs under unstrained and cyclic stretch for 48 h. The hMSCs in **(O)** were treated with ROCK inhibitor, Y27632, before cyclic stretch. The direction of UCS was in the white double-headed arrow direction. **(P)** Mean fluorescence intensity (MFI) of F-actin in **(M–O)**. Significant differences were noted by ****P < 0.0001. ns signifies *P* > 0.05. *n* = 4. **(Q–S)** Distribution analysis of the angle between the direction of nuclear long axis and UCS. The angle is represented by θ'. Every 10° is a range. Numbers of nuclei measured orientation angle in each group were 739, 686, and 520, respectively. The nuclei from four biological replicates were counted together. **(T)** Population distribution of hMSCs nuclear aspect ratio (NAR) in unstrained, 1 Hz + 8% and 1 Hz + 8% + Y27632 condition for 48 h. Blue lines indicate the median of data set, and red lines indicate quartiles. Numbers of nuclei measured NAR in each group were same as in **(Q–S)**. Significant differences were noted by ****P < 0.0001. ns signifies *P* > 0.05.

hMSCs were first cultured on gelatin-coated PDMS chambers for 24 h prior to applying UCS. It can be seen that cells were well attached and spread on the substrate (Figures 2A–D). Then hMSCs were subjected to UCS for 48 h with different frequency and magnitude. The number of cells increased after 48 h stretching. While hMSCs in unstrained group randomly distributed on the substrate as expected (Figure 2E), cellular realignment was obvious at both frequency of 8% magnitude (Figures 2G,H), and the alignment of hMSCs was more perpendicular to the direction of UCS at higher frequency. However, the arrangement of hMSCs, which subjected to 0.5 Hz at 4% magnitude, remained random (Figure 2F).

To quantify the cellular realignment under UCS, the angle θ between the elongated direction of cell and UCS was measured using ImageJ software, statistical analysis then was performed. It was seen that the occurrence frequency shows a uniform distribution without external forces (Figure 2I). Similarly, there was no preferred direction for the 0.5 Hz + 4% group (Figure 2J). As the stretching magnitude increased to 8%, 66% of hMSCs have the alignment angle θ in the range of 70–90° (Figure 2K). With the stretching frequency increased to 1 Hz, the percentage of hMSCs angle range from 70 to 90° was increased to 76% (Figure 2L). In particular, the alignment angle in the range of 80–90° has the highest occurrence frequency (more than 50%). These results indicate that when the stretching magnitude doubled, cell arrangement changed from chaotic to orderly, and cell population underwent an obvious “phase transition," the percentage of cells aligned in the range of 80–90° increased to 3.5 times (Figures 2J,K). With the stretching frequency increased from 0.5 to 1 Hz, the percentage of cell angles ranging from 80 to 90° was further increased to 1.3 times (Figures 2K,L). The order of cell arrangement was increased after doubled frequency, whereas nothing essential has changed. Our results suggest that the realignment of hMSCs was triggered beyond a critical stretching magnitude and influenced by the frequency of UCS.

To further investigate the effect of UCS on the morphology of hMSCs, F-actin was labeled with fluorescent phalloidin, which can specifically bind to the actin stress fibers. As seen in phase-contrast micrographs of stained hMSCs, the direction of actin stress fibers was perpendicular to the direction of UCS under 1 Hz + 8% (Figures 2M,N). Mean fluorescence intensity (MFI) of F-actin fibers with cyclic stretching was significantly higher than those in unstrained cells (89.09 ± 6.34 vs. 112.64 ± 5.28, *P* < 0.0001; Figure 2P).

At the same time, the long axis direction (Figures 2Q,R) and the aspect ratio (Figure 2T) of the nuclei were measured using ImageJ software to investigate the effect of UCS on nuclear deformation. In the unstrained group, the distribution of long axis of nuclei is non-directional (Figure 2Q). While the long axis of nuclei in 1 Hz + 8% UCS tended to align perpendicularly to the stretching direction (Figure 2R). As shown in the violin diagram, the mean value of nuclear aspect ratio (NAR) increased from 1.45 to 1.53 after UCS for 48 h, and this change is statistically significant (1.45 ± 0.23 vs. 1.53 ± 0.23, *P* < 0.0001; Figure 2T).

### 3.2. UCS Inhibits Cell Proliferation

**Figure 3:**
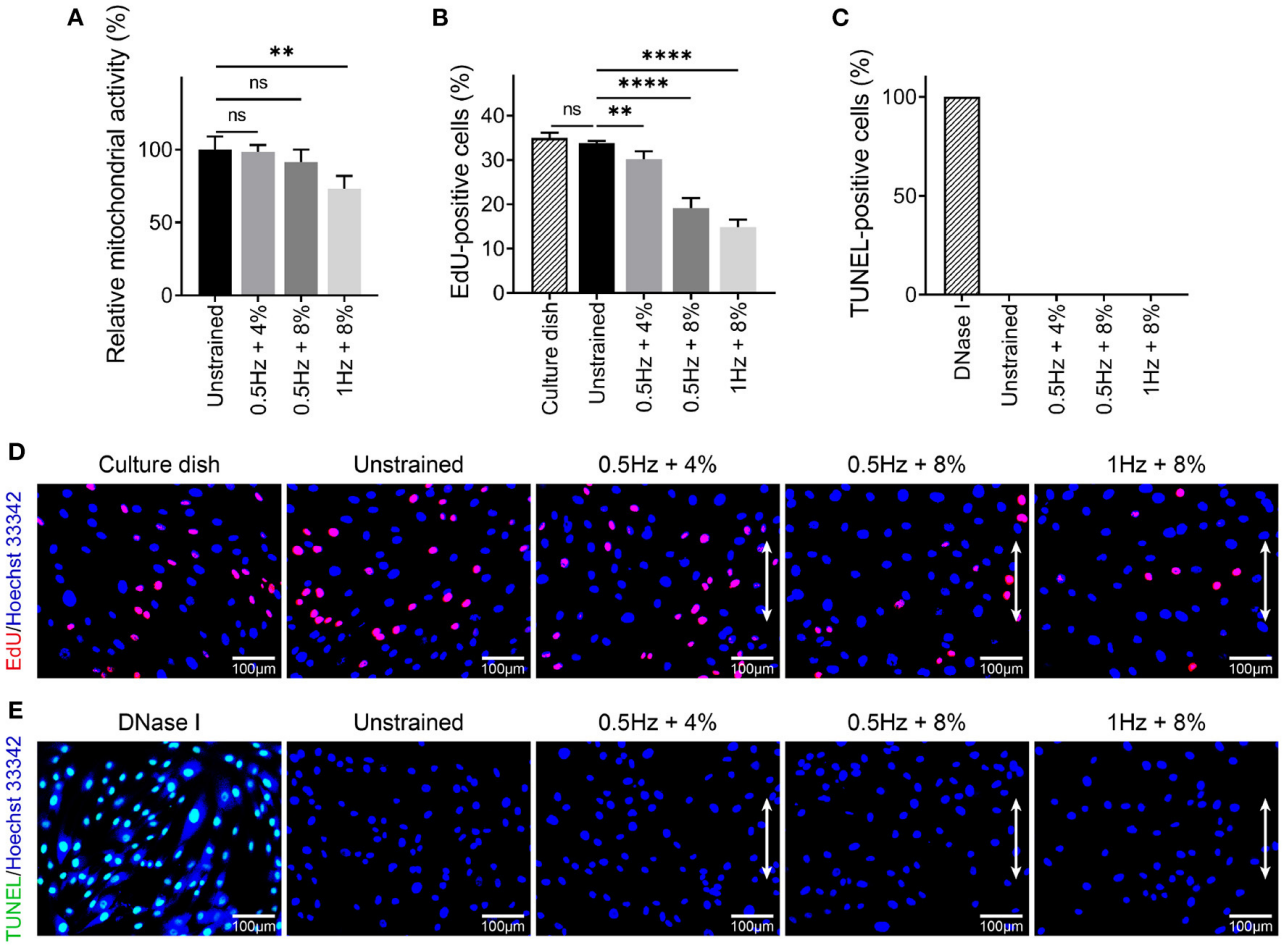
**(A)** Analysis of relative mitochondrial activity of hMSCs in different mechanical conditions. Statistical significance was noted by **P < 0.01. ns signifies *P* > 0.05. *n* = 4. **(B,D)** Quantitative analysis and representative fluorescence images of EdU-positive cells in culture dish and different cyclic stretch. Proliferating cells were labeled with EdU (red) and nuclei were stained with Hoechst 3,3342 (blue). White double-headed arrows indicate the direction of stretching. Statistical significance was noted by **P < 0.01 and ****P < 0.0001. ns signifies *P* > 0.05. *n* = 4. **(C,E)** Quantitative analysis and representative fluorescent images of TUNEL-positive cells under DNase I treatment and different cyclic stretch. Apoptotic cells were labeled with TUNEL (green) and nuclei were stained with Hoechst 33342 (blue). White double-headed arrows indicate the direction of stretching. *n* = 4.

To examine the effect of different stretching amplitude and frequency on cell proliferation and apoptosis, MTT, EdU, and TUNEL assay were performed after cyclic stretching for 48 h. Compared with unstrained group, the relative mitochondrial activity of hMSCs was decreased significantly for the 1 Hz + 8% group (100 ± 8.79% vs. 73.07 ± 8.82%, *P* < 0.01), whereas there was no statistical difference in the other two groups (100 ± 8.79% vs. 98.32 ± 4.81%, *P* > 0.05; 100 ± 8.79% vs. 91.47 ± 8.55%, *P* > 0.05; Figure 3A). EdU staining was used to count the percentage of proliferating cells (Figure 3D). Compared to the unstrained group, all of these three UCS groups showed significant decrease in the percentage of EdU-positive cells (33.79 ± 0.55% vs. 30.20 ± 1.77%, *P* < 0.01; 33.79 ± 0.55% vs. 19.18 ± 2.25%, *P* < 0.0001; 33.79 ± 0.55% vs. 14.83 ± 1.71%, *P* < 0.0001; Figures 3B,D). There was no significant difference in the percentage of EdU-positive cells between in culture dish and in unstrained condition (34.97 ± 1.23% vs. 33.79 ± 0.55%, *P* > 0.05). TUNEL assay were performed to detect apoptosis after cyclic stretching. hMSCs treated with DNase I were used as positive control (Figure 3E). No TUNEL-positive cells were detected in unstrained and UCS groups (Figures 3C,E). These results indicated that the suppressed cell proliferation was correlated with the magnitude and frequency and UCS had no obvious effect on apoptosis. Existing research has shown that there is a relationship between cell elongation and lower proliferation rate (Roca-Cusachs et al., 2008; Thakar et al., 2009; Versaevel et al., 2012; Lv et al., 2018). Specifically, cell elongation induced either by external forces or topography of substrate is associated with chromatin condensation, decreased DNA synthesis, and lower proliferation rate (Lv et al., 2018).

### 3.3. UCS Promotes Expression of Marker Genes Associated With Osteogenesis of hMSCs

**Figure 4:**
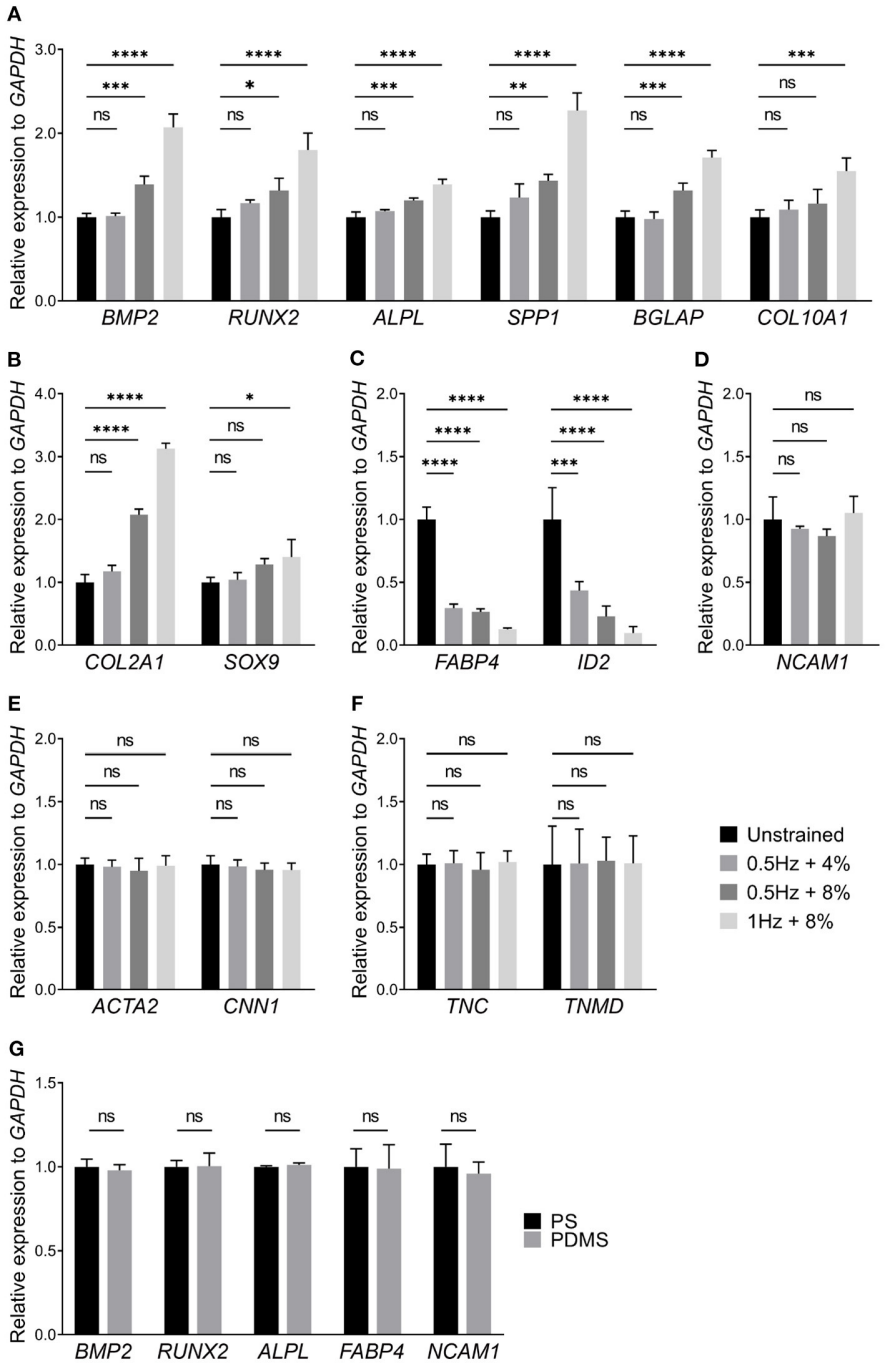
Relative expression levels of **(A)** osteogenic, **(B)** chondrogenic, **(C)** adipogenic, **(D)** neurogenic, **(E)** myogenic, and **(F)** tenogenic marker genes of hMSCs subjected to UCS for 48 h were shown above, respectively. The application of 1 Hz at 8% stretch significantly increased the expression of osteogenic **(A)** and chondrogenic **(B)** marker genes. However, the expression of adipogenic marker genes, *FABP4* and *ID2*, were decreased at three kinds of UCS groups **(C)**. The expression of osteogenic and chondrogenic marker genes between unstrained and 0.5 Hz + 4% strain group showed no change. There were no significant changes in relative expression of genes associated with neurogenesis, myogenesis, and tenogenesis between different mechanical groups **(D–F)**. **(G)** Relative expression of several differentiation marker genes of hMSCs cultured on polystyrene (PS) culture dishes and PDMS chambers for 48 h. No significant changes were found in relative expression of marker genes between PS and PDMS substrates. Significant differences were noted by *P < 0.05, **P < 0.01, ***P < 0.001, and ****P < 0.0001. ns signifies *P* > 0.05. *n* = 4.

In addition to cell morphology and proliferation, cyclic stretching can also influence differentiation of stem cells. Cell lineage was extrapolated by marker genes expression. Specifically, we examined how UCS loading conditions affect the marker genes expression of hMSCs for osteogenesis, chondrogenesis, adipogenesis, neurogenesis, myogenesis, and tenogenesis (Figures 4A–F). To exclude the effect of PDMS substrate on hMSCs differentiation, we compared a part of marker genes expression of hMSCs on polystyrene (PS) culture dishes (704002, NEST) and PDMS chambers through RT-qPCR. There were no significant changes on relative expression between PS and PDMS substrates (Figure 4G). Comparing with the unstrainted group, the expression level of osteogenic marker genes such as *BMP2* (1.39 ± 0.10), *RUNX2* (1.32 ± 0.15), *ALPL* (1.20 ± 0.03), *SPP1* (1.43 ± 0.08), and *BGLAP* (1.32 ± 0.09) were increased when hMSCs were subjected to 8% cyclic stretching at 0.5 Hz for 48 h (Figure 4A). As the stretching frequency changed from 0.5 to 1 Hz, the relative expression level of all tested osteogenic marker genes became even higher (*BMP2*: 2.07 ± 0.16; *RUNX2*: 1.80 ± 0.19; *ALPL*: 1.39 ± 0.06; *SPP1*: 2.27 ± 0.21; *BGLAP*: 1.71 ± 0.09; *COL10A1*: 1.55 ± 0.16). It was also seen that the cyclic stretching promotes the expression of both *COL2A1* and *SOX9*, which are known as chondrogenic marker genes. In particular, the change in the expression of *COL2A1* (3.13 ± 0.09) was significant under 1 Hz + 8% cyclic stretching. The results in Figures 4A,B also indicate that UCS with 0.5 Hz at 4% magnitude has rather small effects on osteogenesis and chondrogenesis. Considering these mechanical conditions, the application of 1 Hz at 8% stretching amplitude was found to be the most favorable condition for osteogenic and chondrogenic differentiation.

It has been established that osteogenic and adipogenic differentiations are opposite directions of differentiation (Nuttall and Gimble, 2004; Chen et al., 2016). It is expected that the expression of adipogenic marker genes, *FABP4* and *ID2*, were decreased in all UCS conditions (Figure 4C). Moreover, gene markers for other differentiation directions were detected too, specifically neurogenic (Figure 4D), myogenic (Figure 4E), and tenogenic (Figure 4F) marker genes. However, there was no statistical significance among those groups. Thus, our results indicated that UCS promotes expression of marker genes associated with osteogenesis of hMSCs in a stretch frequency and amplitude-dependent manner, while it inhibits adipogenic marker genes expression in both frequency and amplitude.

### 3.4. UCS Increases Openness of the Gene Loci Associated With Morphogenesis and Osteogenesis

**Figure 5:**
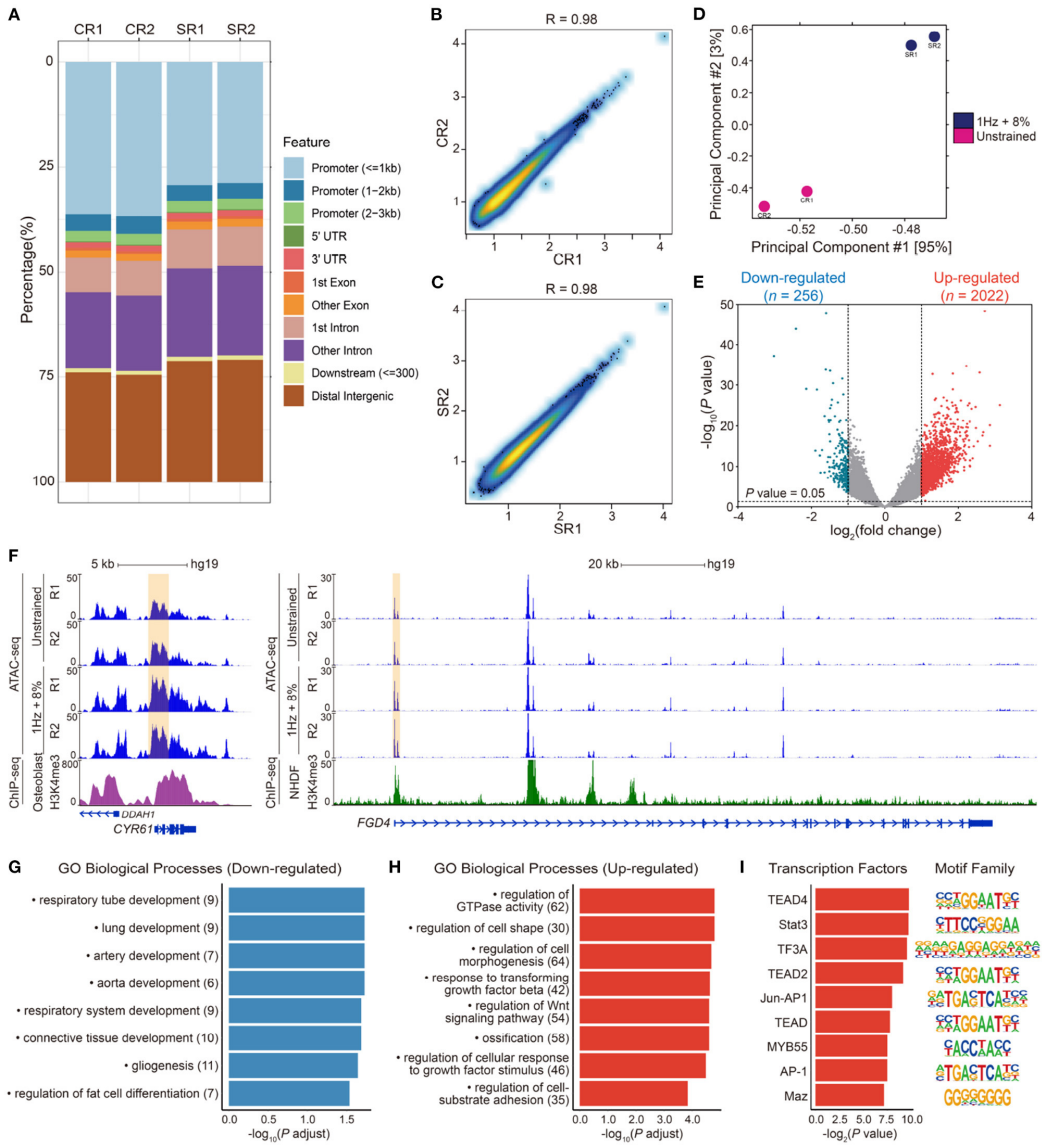
**(A)** Peaks feature distribution of ATAC-seq samples in unstrained (CR1, CR2) and 1 Hz + 8% stretching (SR1, SR2) groups. More than 25% peaks were enriched in promoter regions in each sample. **(B,C)** Pearson correlation analysis of replicate samples ATAC-seq data in unstrained (CR1, CR2) and 1 Hz + 8% (SR1, SR2) groups. There was strong correlation between two replicates in both conditions. **(D)** Principal component analysis (PCA) of chromatin accessibility after cyclic stretching in two dimensions. The red dots indicate two unstrained samples (CR1, CR2), and two blue dots indicate 1 Hz + 8% stretching samples (SR1, SR2). **(E)** Volcano plots analysis of genes with different openness chromatin sites after 1 Hz + 8% cyclic stretch. A total of 2278 gene loci with log_2_(fold change) > 1 or log_2_(fold change) < -1, were altered significantly based on *P* value < 0.05. Among them, 2022 red dots stand for upregulate gene loci, and 256 blue dots stand for downregulate gene loci. Dotted lines in *x*-axis stand for log_2_(fold change) = ± 1 and dotted line in *y*-axis stands for *P* value = 0.05. **(F)** Normalized epigenetic profiles at *CYR61* (ch1: 86046444-86049648) and *FGD4* (ch12: 32655041-32798984) loci in unstrained and 1 Hz + 8% stretching groups. H3K4me3 ChIP-seq profiles of human osteoblasts at *CYR61* loci and normal human dermal fibroblasts (NHDF) at *FGD4* loci were shown below. The yellow highlighted regions represented promoter regions of genes. ChIP-seq data were downloaded from Encode (wgEncodeBroadHistoneOsteoH3k04me3 and wgEncodeBroadHistoneNhdfadH3k4me3Std). All signals were modified from the WashU epigenome browser. **(G,H)** Gene ontology (GO) biological process enrichment of 256 downregulated **(G)** and 2022 upregulated **(H)** gene loci in differentially chromatin accessibility. Pathways related to cell morphogenesis, adhesion, and differentiation were selected to complete the figure. All terms are significantly enriched with *P* value < 0.05. *X*-axis indicates -log_10_(*P* adjust) and *y*-axis indicates enriched terms. The number of genes enriched in GO terms is noted in parentheses after each corresponding term. **(I)** Transcription factors enrichment in promoter of genes which presented in upregulated GO terms (left) and their motifs (right). *Y*-axis indicates predicted transcription factors.

To investigate the effect of cyclic stretching on chromatin accessibility of hMSCs, we compared accessible site between unstrained and 1 Hz + 8% groups using ATAC-seq. Over 25% of peaks in each sample were enriched at promoter regions (Figure 5A). Transcription starting site (TSS) enrichment and Pearson correlation analysis of the replicate samples showed high-quality data and strong correlation between replicates (Figures 5B,C). Principal components analysis (PCA) revealed that the difference of chromatin accessibility between the unstrained and 1 Hz + 8% groups in two dimensions (Figure 5D). There were a total of 2,278 gene loci that had the accessibility change with *P* < 0.05 and fold change > 2. While decreased accessibility was found in 256 gene sites, the accessibility of 2,022 gene sites was increased (Figure 5E).

Moreover, we also associated peaks with their nearby genes. It was found that peak signaling increased in promoter regions of genes associated with osteogenesis and morphogenesis, such as *CYR61* and *FGD4* (Figure 5F). Focusing on these down- and upregulated loci, the related biological processes were analyzed by gene ontology (GO). GO enrichment analysis indicated that gene loci with decreased chromatin accessibility were related to respiratory tube, lung, artery, and aorta development (Figure 5G). There were seven downregulated genes associated with regulation of fat cell differentiation (*INSIG1*, *DUSP10*, *HTR2A*, *PPARD*, *ID2*, *HES1*, and *WNT5B*). Among them, downregulation of *ID2* was validated by RT-qPCR results (Figure 4C). Gene loci with increased chromatin accessibility were mainly associated with regulation of GTPase activity, cell morphogenesis, cell–substrate adhesion, and ossification (Figure 5H). It has been found that small GTPase Rho and Rho-kinase affect the rearrangement of endothelial cells stress fibers during mechanical stretching (Kaunas et al., 2005). To verify the effect of GTPase activity on cell morphology and nuclear deformation in this UCS platform, hMSCs were incubated in culture media with 10 μM Y27632 to inhibit Rho-kinase activity. After Y27632 treatment, the F-actin became random after UCS (Figure 2O), and the MFI decreased significantly compared with 1 Hz + 8% group (112.6 ± 5.3 vs. 88.5 ± 6.7, *P* < 0.0001; Figure 2P). However, there was no significant change in MFI between unstrained and 1 Hz + 8% + Y27632 group (89.09 ± 6.34 vs. 88.54 ± 6.67, *P* > 0.05; Figure 2P). The distribution of nuclei long axis became non-directional again under UCS with Y27632 (Figure 2S). Compared to 1 Hz + 8% group, the NAR decreased significantly after treatment with Y27632 and UCS (1.53 ± 0.23 vs. 1.44 ± 0.19, *P* < 0.0001; Figure 2T). However, no significant change of NAR was found between unstrained and 1 Hz + 8% + Y27632 group (1.45 ± 0.23 vs. 1.44 ± 0.19, *P* > 0.05; Figure 2T). Y27632 treatment seemed to counteract the effect of UCS on cell morphology and nuclear deformation. Moreover, three osteogenic marker genes (Figure 4A), *BMP2*, *RUNX2*, and *BGLAP*, were also found in ossification of upregulated GO biological processes.

Additionally, signaling pathways associated with osteogenesis were enriched in upregulated GO biological processes, such as TGF-β, and Wnt signaling pathways. To identify potential transcription factors (TFs) involved in cyclic stretch of hMSCs, elevated ATAC-seq enrichment at promoter regions of genes included in eight main groups in Figure 5H were used to predict TFs occupancy (Figure 5I). Among these TFs, TEAD/2/4 had been reported to interact with YAP, which is a sensor and mediator of mechanical cues during mechanotransduction (Zhao et al., 2008; Dupont et al., 2011). STAT3 could induce expression of genes implicated in anti-proliferation and osteogenic differentiation (Blanchard et al., 2009). AP-1 binding site was present at promoter regions of *OPN* (*SPP1*), *IL6*, and *TGFB1* gene sites, all of these genes were contribute to osteogenesis (Erices et al., 2002; Wu et al., 2016; Bailey et al., 2017). Thus, our results indicated that UCS increases the openness of gene loci not only associated with regulation of cell morphogenesis but also with osteogenesis.

## 4. Discussion

### 4.1. Actin Stress Fibers Realignment in UCS

It is well established that external mechanical stimuli influence a multitude of cellular behaviors including cell adhesion (Na et al., 2008), orientation (Geiger et al., 2006), proliferation (Nam et al., 2015; Paul et al., 2017), and differentiation (Jagodzinski et al., 2004; Qi et al., 2008; Kang et al., 2012; Carroll et al., 2017). Sensing external mechanical forces starts from cell–matrix interactions that are mediated by cell adhesion receptors like integrins. Through the formation of focal adhesions, the integrins and surface proteins of matrix are linked to the actin cytoskeleton (Fletcher and Mullins, 2010). MSCs can “feel” the surrounding mechanical environment using the contractile forces generated by actin–myosin networks, and the feedback from the environment can then lead to modification of cytoskeletal features such as stress fibers and changes in cell shape accordingly (Geiger et al., 2006). In this study, we investigated how hMSCs responded to UCS with varying magnitude and frequency. Specifically, the observations from cell morphology study suggest that the reorientation of hMSCs was triggered when a stretch magnitude threshold was reached. The alignment direction was perpendicular to the stretching direction, and the degree of alignment increased with the magnitude and frequency of UCS (Figures 2I–L). Similar cell reorientation phenomena were reported in the literature (Hamilton et al., 2004; Park et al., 2004; Kurpinski et al., 2006; Chen et al., 2008; Carroll et al., 2017). For instance, Park et al. (2004) investigated differentiation effects of cyclic equiaxial and uniaxial strains on MSCs. After 1 day under 10% cyclic uniaxial strain at 1 Hz, MSCs orientated themselves perpendicular to the strain direction and the stress fibers aligned in the same direction. Cell reorientation in response to cyclic stretch is thought to be an escape mechanism to avoid stress (Buck, 1980; Kaunas et al., 2005; Jungbauer et al., 2008). When cells are exposed to stretching environment, the actin filaments remodel to reorient and alter the transmission path of stress to maintain mechanical homeostasis, and eventually cells align their long axes in the direction of the minimal substrate deformation.

Actin stress fibers play a critical role in the process of cell reorientation induced by mechanical stress. Hayakawa et al. (2000) found the realignment of stress fibers preceded reorientation of cells. Rat aortic smooth muscle cells were subjected to cyclic stretch with an amplitude of 20% at 1 Hz. After 15 min of cyclic stretching, actin stress fibers were aligned obliquely to the direction of stretching with angles of 50–70°. Then, most of cells were reoriented to directions similar with the stress fibers after 1–3 h of stretching. The research demonstrated that there are a sequence of events from actin stress fibers realignment to cell reorientation. Before stretching, the actin filaments are subjected in isotropic forces, however, UCS leads to increased tension of stress fibers along the stretching direction and causes actin filaments paralleled to the stretching direction to break and depolymerize into pieces (Hayakawa et al., 2001). As a result, these filaments cannot mature to stress fibers bundles in the direction parallel to stretching. Actin filaments then reassemble along the direction of minimum deformation, which is the direction perpendicular to stretching, and finally stabilize to mature stress fibers bundles (Kaunas et al., 2005; Geiger et al., 2006). As observed in our studies, actin filaments of hMSCs subjected to 8% + 1 Hz were rearranged perpendicular to the stretching direction and thicken (Figures 2M–P). This observation also confirms that actin filaments are much likely to assemble in the direction of minimum deformation.

In addition to being directly induced by mechanical stretching, the rearrangement of stress fibers could also be mediated by Rho GTPase signaling (Kaunas et al., 2005; Lee et al., 2010). Hayakawa et al. (2001) found stretch-induced stress fibers rearrangement and cell reorientation were inhibited by Rho GTPase inhibitor botulinum C3 transferase. However, Roland et al. investigated the cooperative effects of Rho and mechanical stretch on stress fiber organization. They found Y27632-treated endothelial cells under uniaxial stretch formed stress fibers parallel to stretch direction, and stress fibers then bundled together to form thick fibers (Kaunas et al., 2005; Lee et al., 2010). Rho GTPase activity may play a critical role in determining the direction and extent of stress fibers orientation induced by mechanical stretch. This is consistent with our first term in upregulated GO biological process, regulation of GTPase activity, which is involved in cyclic stretch-induced rearrangement of stress fibers (Figure 5H). Our results also show that, when the hMSCs were treated with Y27632, the USC-induced stress fiber rearrangement and nuclear deformation were inhibited (Figures 2M–T). In recent years, more and more studies have found that cell reorientation not only depends on the arrangement of stress fibers but also is controlled by focal adhesion, which link cytoskeleton with substrate (Geiger et al., 2009; Chen et al., 2012a). Chen et al. investigated the phenomena of cell reorientation on substrates induced by uniaxial cyclic stretch. By using a minimal model of cellular mechanosensing system, they found focal adhesions were destabilized by cyclic stretch through reducing catch bonds lifetime, which result in focal adhesions slide or relocation and then cause the associated stress fibers rotate almost perpendicular to the stretching direction (Chen et al., 2012a). Experimentally, Greiner et al. (2013) investigated cell shape and actin stress fibers formation and focal adhesion alignment in spreading fibroblasts, which exposed to cyclic tensile strain, and demonstrated that periodic substrate forces affect contractile actin cytoskeleton formation and related focal adhesion sites. Regulation of cell–substrate adhesion was also enriched in our upregulated GO biological process (Figure 5H). Associated with our results, we conclude that cell orientation induced by cyclic stretch correlates with the actin stress fibers arrangement as well as cell-substrate adhesions regulation.

### 4.2. Osteogenic Marker Genes Expression in UCS

As the nucleus is surrounded by the cytoskeleton, external mechanical forces that lead to the remodeling of cytoskeleton can naturally propagate to the nuclear envelop and possibly influence the organization of chromatin (Arnsdorf et al., 2010; Ramdas and Shivashankar, 2015). Furthermore, an emerging understanding is that mechanical signals that transmit to the nucleus can activate different gene expression programs and regulate transcription factors (Kirby and Lammerding, 2018). It is of great interest to understand the effects of external mechanical forces on hMSCs differentiation owing to the plural potent nature of hMSCs. We tested the expression levels of the marker genes of hMSCs for osteogenesis, chondrogenesis, adipogenesis, neurogenesis, myogenesis, and tenogenesis using RT-qPCR (Figures 4A–F). The results show that the levels of multiple marker genes expression for osteogenesis increased when applied magnitude and frequency of UCS increased, while the application of UCS reduced the expression levels of marker genes for adipogenesis. Specifically, the osteogenic marker genes with increased expression level included *BMP2*, *RUNX2*, *ALPL*, *SPP1*, *BGLAP*, and *COL10A1*. These findings agree with reports from existing studies (Chen et al., 2008; Carroll et al., 2017). For example, Carroll et al. (2017) demonstrated that, when hMSCs encapsulated in fibrin hydrogels were under cyclic tensile strain (10% strain at 0.5 Hz) only, higher levels of expression were obtained for *BMP2*, *RUNX2*, and *ALPL* after 7 days. The study also showed that the level of expression was suppressed for the adipogenic marker *LPL*. In addition, it has also been reported that the cyclic tensile strain promoted chondrogenic differentiation (Chen and Wu, 2019), neurogenic differentiation (Leong et al., 2012), and tenogenic differentiation (Morita et al., 2019). However, we only observed that the expression levels of marker genes for chondrogenesis, *COL2A1* and *SOX9*, increased in a magnitude-frequency dependent manner. As there are multitude factors affecting the differentiation of hMSCs, the discrepancy may be attributed to the differences in culture media, substrates, and duration, magnitude, and frequency of loading conditions.

### 4.3. Nuclear Deformation and Chromatin Remodeling in UCS

A number of experiments have shown that external forces physically transfer from the ECM to the nucleus and have identified nucleus morphological changes under mechanical stress (Wang et al., 2009, 2018; Shivashankar, 2011). In addition to external mechanical forces, differential substrate can also cause nuclear deformation, such as nanofibers (Heo et al., 2011; Nathan et al., 2011), micropillar array (Liu and Ding, 2020), and micropattern (Versaevel et al., 2012). By using a combination of micro-manipulation tools, Versaevel et al. (2012) found that nucleus deformed by a high level of compressive forces of actin filaments on both sides during cell elongation. The shape and structure of nuclei are affected by large-scale cell shape changes. Their results indicated that actin filaments are essential for the mechanical process of nuclear remodeling. Prior to this literature, Khatau et al. (2009) have reported the presence of actin cap, a dome-like actin structure, covers the top of the nucleus, and they demonstrated that actin cap can regulate the nuclear shape. These above studies showed that there is a mechanistic coordination between cytoskeleton and nuclear shape. By labeling F-actin with fluorescent phalloidin and staining nuclei with Hoechst 33342, we observed thicker stress fiber bundles appear and perpendicular to the tensile direction after 1 Hz + 8% cyclic stretch for 48 h (Figures 2M–P). Meanwhile, the long axis of most nuclei is reoriented perpendicular to the stretching direction (Figures 2Q,R) and a significant increase in nuclear aspect ratio was observed (Figure 2T). We noticed that the distribution and average (θ_*average*_ = 75.45°, $\theta_{average}^{\prime }$ = 64.19°) of the long axes orientation between cells and nuclei are similar in 1 Hz + 8% group. According to these previous studies of spatial coordination between cellular and nuclear shape (Versaevel et al., 2012), we speculate that one possibility for nuclear deformation induced by UCS is due to lateral actin filaments compression. Another possibility is stress fibers tension causes nuclear deformation, which transmitted to the surface of nucleus through LINC (Linker of Nucleoskeleton and Cytoskeleton) complex (Athirasala et al., 2017).

It is becoming recognized that the nucleus mechanical properties play a vital role in chromatin remodeling and gene expression. Nuclear deformation often results in changes in chromatin organization and genome function (Thomas et al., 2002; Lanctot et al., 2007). Maniotis et al. (1997) proposed that changes in cell morphology are transmitted into gene expression through cytoskeleton. This hypothesis implies that cytoskeleton regulate nuclear shape by applying external force in response to the change in cell shape. Treatment with inhibitors and RNA interference of cytoskeleton in *Drosophila* cells, Ramdas and Shivashankar demonstrated that modulations of cytoskeleton assembly exerts mechanical control on nuclear morphology and chromatin remodeling (Ramdas and Shivashankar, 2015). It is critical to understand mechanisms that enable gene activation and regulation of transcription due to mechanical signals transmitted to the nucleus. One of mechanisms that have been proposed is that external forces can induce deformation of chromatin and alter transcription factor activity (Kirby and Lammerding, 2018). For instance, Heo and colleagues has demonstrated that dynamic tensile loading (DL) regulates chromatin organization in MSCs, and the rate and degree of condensation depends on the frequency and duration of mechanical loading (Heo et al., 2016b). Furthermore, it is thought that external mechanical stimulus, which induces anisotropic forces of cytoskeleton, causes histone acetylation, H3K4 methylation, and consequently activates transcription (Lv et al., 2018). However, the openness of mechanosensitive genes in mechanical stress-induced stem cells differentiation is still unknown. To this end, we examined the accessibility change of chromatin and assessed the openness of the gene loci via ATAC-seq. In terms of the whole accessibility changes of chromatin, the number of more open gene loci was greater than the closed after UCS (Figure 5E). Gene loci with increased chromatin accessibility were mainly associated with regulation of cell morphogenesis, cell–substrate adhesion, and ossification (Figure 5H). For instance, three osteogenic marker genes (Figure 4A), *BMP2*, *RUNX2*, and *BGLAP*, were found in ossification of upregulated GO biological processes. There were also seven downregulated genes associated with regulation of fat cell differentiation (*INSIG1*, *DUSP10*, *HTR2A*, *PPARD*, *ID2*, *HES1*, *WNT5B*). Among them, downregulation of *ID2* was validated by RT-qPCR results (Figure 4C). Moreover, signaling pathways associated with osteogenesis were also included in the upregulated GO biological processes. Analysis was performed to identify potential TFs involved in cyclic stretch of hMSCs. The results demonstrated that TFs associated with genes contributing to osteogenesis were among those TFs predicted (Figure 5I). Our findings clearly indicated that UCS increased the openness of gene loci associated with regulation of cell morphogenesis and osteogenesis as well as the corresponding transcription activities.

While the increased accessibility of some chromatin regions due to UCS were demonstrated in this study, there are still many remaining questions regarding how the external forces induce chromatin deformation and how changes in chromatin organization affect the openness of gene loci and the regulation of transcription activities. Innovative technologies such as fluorescence lifetime imaging and gene-sequencing for single cell analysis enable us to study chromatin reorganization in live cells and functional regulatory elements (Song et al., 2020). With the advancement in technologies, future research exploring these questions will contribute to our understanding in regulating cell fate and function.

## Data Availability Statement

The datasets presented in this study can be found in online repositories. The names of the repository/repositories and accession number(s) can be found below: GEO accession GSE166410, https://www.ncbi.nlm.nih.gov/geo/query/acc.cgi?acc=GSE166410.

## Author Contributions

HL, XS, and KY conceived the experiment. DZ performed cells culture, seeding, cell morphology, proliferation, and apoptosis assay. DZ and RZ finished RT-qPCR and ATAC-seq experiments. RZ conducted the bioinformatic analyses. All authors have read, wrote, and approved the manuscript.

## Conflict of Interest

The authors declare that the research was conducted in the absence of any commercial or financial relationships that could be construed as a potential conflict of interest.

## References

[B1] Arnsdorf E. J. Tummala P. Castillo A. B. Zhang F. Jacobs C. R. (2010). The epigenetic mechanism of mechanically induced osteogenic differentiation. J. Biomech. 43, 2881–2886. 10.1016/j.jbiomech.2010.07.033 20728889 PMC2975768

[B2] Arnsdorf E. J. Tummala P. Kwon R. Y. Jacobs C. R. (2009). Mechanically induced osteogenic differentiation-the role of rhoa, rockii and cytoskeletal dynamics. J. Cell Sci. 122(Pt 4), 546–553. 10.1242/jcs.036293 19174467 PMC2714434

[B3] Athirasala A. Hirsch N. Buxboim A. (2017). Nuclear mechanotransduction: sensing the force from within. Curr. Opin. Cell Biol. 46, 119–127. 10.1016/j.ceb.2017.04.004 28641092

[B4] Bailey S. Karsenty G. Gundberg C. Vashishth D. (2017). Osteocalcin and osteopontin influence bone morphology and mechanical properties. Ann. N.Y. Acad. Sci. 1409, 79–84. 10.1111/nyas.13470 29044594 PMC5730490

[B5] Baron R. Kneissel M. (2013). Wnt signaling in bone homeostasis and disease: from human mutations to treatments. Nat. Med. 19, 179–192. 10.1038/nm.3074 23389618

[B6] Blanchard F. Duplomb L. Baud'huin M. Brounais B. (2009). The dual role of il-6-type cytokines on bone remodeling and bone tumors. Cytokine Growth Factor Rev. 20, 19–28. 10.1016/j.cytogfr.2008.11.004 19038573

[B7] Buck R. C. (1980). Reorientation response of cells to repeated stretch and recoil of the substratum. Exper. Cell Res. 127, 470–474. 10.1016/0014-4827(80)90456-5 7379874

[B8] Buenrostro J. D. Giresi P. G. Zaba L. C. Chang H. Y. Greenleaf W. J. (2013). Transposition of native chromatin for fast and sensitive epigenomic profiling of open chromatin, dna-binding proteins and nucleosome position. Nat. Methods 10, 1213–1218. 10.1038/nmeth.2688 24097267 PMC3959825

[B9] Carroll S. F. Buckley C. T. Kelly D. J. (2017). Cyclic tensile strain can play a role in directing both intramembranous and endochondral ossification of mesenchymal stem cells. Front. Bioeng. Biotechnol. 5:73. 10.3389/fbioe.2017.00073 29230389 PMC5712005

[B10] Chaudhuri O. Gu L. Klumpers D. Darnell M. Bencherif S. A. Weaver J. C.. (2016). Hydrogels with tunable stress relaxation regulate stem cell fate and activity. Nat. Mater. 15, 326–334. 10.1038/nmat4489 26618884 PMC4767627

[B11] Chen B. Kemkemer R. Deibler M. Spatz J. Gao H. (2012a). Cyclic stretch induces cell reorientation on substrates by destabilizing catch bonds in focal adhesions. PLoS ONE 7:e48346. 10.1371/journal.pone.0048346 23152769 PMC3495948

[B12] Chen G. Deng C. Li Y. P. (2012b). Tgf-beta and bmp signaling in osteoblast differentiation and bone formation. Int. J. Biol. Sci. 8, 272–288. 10.7150/ijbs.2929 22298955 PMC3269610

[B13] Chen J. Wu X. (2019). Cyclic tensile strain promotes chondrogenesis of bone marrow-derived mesenchymal stem cells by increasing mir-365 expression. Life Sci. 232:116625. 10.1016/j.lfs.2019.116625 31276691

[B14] Chen Q. Shou P. Zheng C. Jiang M. Cao G. Yang Q.. (2016). Fate decision of mesenchymal stem cells: adipocytes or osteoblasts? Cell Death Differ. 23, 1128–1139. 10.1038/cdd.2015.168 26868907 PMC4946886

[B15] Chen Y. J. Huang C. H. Lee I. C. Lee Y. T. Chen M. H. Young T. H. (2008). Effects of cyclic mechanical stretching on the mrna expression of tendon/ligament-related and osteoblast-specific genes in human mesenchymal stem cells. Connect Tissue Res. 49, 7–14. 10.1080/03008200701818561 18293173

[B16] Cheng S. L. Yang J. W. Rifas L. Zhang S. F. Avioli L. V. (1994). Differentiation of human bone marrow osteogenic stromal cells *in vitro*: induction of the osteoblast phenotype by dexamethasone. Endocrinology 134, 277–286. 10.1210/endo.134.1.8275945 8275945

[B17] Dalby M. J. Gadegaard N. Tare R. Andar A. Riehle M. O. Herzyk P.. (2007). The control of human mesenchymal cell differentiation using nanoscale symmetry and disorder. Nat. Mater. 6, 997–1003. 10.1038/nmat2013 17891143

[B18] Day T. F. Guo X. Garrett-Beal L. Yang Y. (2005). Wnt/beta-catenin signaling in mesenchymal progenitors controls osteoblast and chondrocyte differentiation during vertebrate skeletogenesis. Dev. Cell 8, 739–750. 10.1016/j.devcel.2005.03.016 15866164

[B19] Dupont S. Morsut L. Aragona M. Enzo E. Giulitti S. Cordenonsi M.. (2011). Role of yap/taz in mechanotransduction. Nature 474, 179–183. 10.1038/nature10137 21654799

[B20] Engler A. J. Sen S. Sweeney H. L. Discher D. E. (2006). Matrix elasticity directs stem cell lineage specification. Cell 126, 677–689. 10.1016/j.cell.2006.06.044 16923388

[B21] Erices A. Conget P. Rojas C. Minguell J. J. (2002). Gp130 activation by soluble interleukin-6 receptor/interleukin-6 enhances osteoblastic differentiation of human bone marrow-derived mesenchymal stem cells. Exp. Cell Res. 280, 24–32. 10.1006/excr.2002.5627 12372336

[B22] Fletcher D. A. Mullins R. D. (2010). Cell mechanics and the cytoskeleton. Nature 463, 485–492. 10.1038/nature08908 20110992 PMC2851742

[B23] Fu J. Wang Y. K. Yang M. T. Desai R. A. Yu X. Liu Z.. (2010). Mechanical regulation of cell function with geometrically modulated elastomeric substrates. Nat. Methods 7, 733–736. 10.1038/nmeth.1487 20676108 PMC3069358

[B24] Geiger B. Spatz J. P. Bershadsky A. D. (2009). Environmental sensing through focal adhesions. Nat. Rev. Mol. Cell Biol. 10, 21–33. 10.1038/nrm2593 19197329

[B25] Geiger R. C. Taylor W. Glucksberg M. R. Dean D. A. (2006). Cyclic stretch-induced reorganization of the cytoskeleton and its role in enhanced gene transfer. Gene Ther. 13, 725–731. 10.1038/sj.gt.3302693 16437132 PMC4150916

[B26] Greiner A. M. Chen H. Spatz J. P. Kemkemer R. (2013). Cyclic tensile strain controls cell shape and directs actin stress fiber formation and focal adhesion alignment in spreading cells. PLoS ONE 8:e77328. 10.1371/journal.pone.0077328 24204809 PMC3810461

[B27] Hamilton D. W. Maul T. M. Vorp D. A. (2004). Characterization of the response of bone marrow-derived progenitor cells to cyclic strain: implications for vascular tissue-engineering applications. Tissue Eng. 10, 361–369. 10.1089/107632704323061726 15165453

[B28] Hayakawa K. Hosokawa A. Yabusaki K. Obinata T. (2000). Orientation of smooth muscle-derived a10 cells in culture by cyclic stretching: relationship between stress fiber rearrangement and cell reorientation. Zoolog Sci. 17, 617–624. 10.2108/zsj.17.617 18517297

[B29] Hayakawa K. Sato N. Obinata T. (2001). Dynamic reorientation of cultured cells and stress fibers under mechanical stress from periodic stretching. Exp. Cell Res. 268, 104–114. 10.1006/excr.2001.5270 11461123

[B30] Heo S. J. Driscoll T. P. Thorpe S. D. Nerurkar N. L. Baker B. M. Yang M. T.. (2016a). Differentiation alters stem cell nuclear architecture, mechanics, and mechano-sensitivity. Elife 5:e18207. 10.7554/eLife.18207 27901466 PMC5148611

[B31] Heo S. J. Han W. M. Szczesny S. E. Cosgrove B. D. Elliott D. M. Lee D. A.. (2016b). Mechanically induced chromatin condensation requires cellular contractility in mesenchymal stem cells. Biophys. J. 111, 864–874. 10.1016/j.bpj.2016.07.006 27558729 PMC5002070

[B32] Heo S. J. Nerurkar N. L. Baker B. M. Shin J. W. Elliott D. M. Mauck R. L. (2011). Fiber stretch and reorientation modulates mesenchymal stem cell morphology and fibrous gene expression on oriented nanofibrous microenvironments. Ann. Biomed. Eng. 39, 2780–2790. 10.1007/s10439-011-0365-7 21800203 PMC3236508

[B33] Hock J. M. Centrella M. Canalis E. (1988). Insulin-like growth factor-i has independent effects on bone-matrix formation and cell replication. Endocrinology 122, 254–260. 10.1210/endo-122-1-254 3335207

[B34] Huebsch N. Arany P. R. Mao A. S. Shvartsman D. Ali O. A. Bencherif S. A.. (2010). Harnessing traction-mediated manipulation of the cell/matrix interface to control stem-cell fate. Nat. Mater. 9, 518–526. 10.1038/nmat2732 20418863 PMC2919753

[B35] Iyer K. V. Pulford S. Mogilner A. Shivashankar G. V. (2012). Mechanical activation of cells induces chromatin remodeling preceding mkl nuclear transport. Biophys. J. 103, 1416–1428. 10.1016/j.bpj.2012.08.041 23062334 PMC3471483

[B36] Jagodzinski M. Drescher M. Zeichen J. Hankemeier S. Krettek C. Bosch U.. (2004). Effects of cyclic longitudinal mechanical strain and dexamethasone on osteogenic differentiation of human bone marrow stromal cells. Eur. Cells Mater. 7, 35–41. 10.22203/eCM.v007a04 15095254

[B37] Jaiswal R. K. Jaiswal N. Bruder S. P. Mbalaviele G. Marshak D. R. Pittenger M. F. (2000). Adult human mesenchymal stem cell differentiation to the osteogenic or adipogenic lineage is regulated by mitogen-activated protein kinase. J. Biol. Chem. 275, 9645–9652. 10.1074/jbc.275.13.9645 10734116

[B38] Jungbauer S. Gao H. Spatz J. P. Kemkemer R. (2008). Two characteristic regimes in frequency-dependent dynamic reorientation of fibroblasts on cyclically stretched substrates. Biophys. J. 95, 3470–3478. 10.1529/biophysj.107.128611 18515393 PMC2547421

[B39] Kang M. N. Yoon H. H. Seo Y. K. Park J. K. (2012). Effect of mechanical stimulation on the differentiation of cord stem cells. Connect Tissue Res. 53, 149–159. 10.3109/03008207.2011.619284 22149641

[B40] Kaunas R. Nguyen P. Usami S. Chien S. (2005). Cooperative effects of rho and mechanical stretch on stress fiber organization. Proc. Natl. Acad. Sci. U.S.A. 102, 15895–15900. 10.1073/pnas.0506041102 16247009 PMC1276069

[B41] Khatau S. B. Hale C. M. Stewart-Hutchinson P. Patel M. S. Stewart C. L. Searson P. C.. (2009). A perinuclear actin cap regulates nuclear shape. Proc. Natl. Acad. Sci. U.S.A. 106, 19017–19022. 10.1073/pnas.0908686106 19850871 PMC2776434

[B42] Khetan S. Guvendiren M. Legant W. R. Cohen D. M. Chen C. S. Burdick J. A. (2013). Degradation-mediated cellular traction directs stem cell fate in covalently crosslinked three-dimensional hydrogels. Nat. Mater. 12, 458–465. 10.1038/nmat3586 23524375 PMC3633615

[B43] Kilian K. A. Bugarija B. Lahn B. T. Mrksich M. (2010). Geometric cues for directing the differentiation of mesenchymal stem cells. Proc. Natl. Acad. Sci. U.S.A. 107, 4872–4877. 10.1073/pnas.0903269107 20194780 PMC2841932

[B44] Kirby T. J. Lammerding J. (2018). Emerging views of the nucleus as a cellular mechanosensor. Nat. Cell Biol. 20, 373–381. 10.1038/s41556-018-0038-y 29467443 PMC6440800

[B45] Kolf C. M. Cho E. Tuan R. S. (2007). Mesenchymal stromal cells. biology of adult mesenchymal stem cells: regulation of niche, self-renewal and differentiation. Arthritis Res. Ther. 9, 204. 10.1186/ar2116 17316462 PMC1860068

[B46] Kurpinski K. Chu J. Hashi C. Li S. (2006). Anisotropic mechanosensing by mesenchymal stem cells. Proc. Natl. Acad. Sci. U.S.A. 103, 16095–16100. 10.1073/pnas.0604182103 17060641 PMC1637542

[B47] Lanctot C. Cheutin T. Cremer M. Cavalli G. Cremer T. (2007). Dynamic genome architecture in the nuclear space: regulation of gene expression in three dimensions. Nat. Rev. Genet. 8, 104–115. 10.1038/nrg2041 17230197

[B48] Lee C. F. Haase C. Deguchi S. Kaunas R. (2010). Cyclic stretch-induced stress fiber dynamics-dependence on strain rate, rho-kinase and mlck. Biochem. Biophys. Res. Commun. 401, 344–349. 10.1016/j.bbrc.2010.09.046 20849825

[B49] Leong W. S. Wu S. C. Pal M. Tay C. Y. Yu H. Li H.. (2012). Cyclic tensile loading regulates human mesenchymal stem cell differentiation into neuron-like phenotype. J. Tissue Eng. Regen. Med. 6(Suppl. 3):s68–s79. 10.1002/term.1548 22777815

[B50] Li C. Vepari C. Jin H. J. Kim H. J. Kaplan D. L. (2006). Electrospun silk-bmp-2 scaffolds for bone tissue engineering. Biomaterials 27, 3115–3124. 10.1016/j.biomaterials.2006.01.022 16458961

[B51] Liu R. Ding J. (2020). Chromosomal repositioning and gene regulation of cells on a micropillar array. ACS Appl. Mater. Interfaces 12, 35799–35812. 10.1021/acsami.0c05883 32667177

[B52] Lv L. Tang Y. Zhang P. Liu Y. Bai X. Zhou Y. (2018). Biomaterial cues regulate epigenetic state and cell functions-a systematic review. Tissue Eng. Part B Rev. 24, 112–132. 10.1089/ten.teb.2017.0287 28903618

[B53] Maniotis A. J. Chen C. S. Ingber D. E. (1997). Demonstration of mechanical connections between integrins, cytoskeletal filaments, and nucleoplasm that stabilize nuclear structure. Proc. Natl. Acad. Sci. U.S.A. 94, 849–854. 10.1073/pnas.94.3.849 9023345 PMC19602

[B54] Marfia G. Navone S. E. Di Vito C. Ughi N. Tabano S. Miozzo M.. (2015). Mesenchymal stem cells: potential for therapy and treatment of chronic non-healing skin wounds. Organogenesis 11, 183–206. 10.1080/15476278.2015.1126018 26652928 PMC4879897

[B55] McBeath R. Pirone D. M. Nelson C. M. Bhadriraju K. Chen C. S. (2004). Cell shape, cytoskeletal tension, and rhoa regulate stem cell lineage commitment. Dev. Cell. 6, 483–495. 10.1016/S1534-5807(04)00075-9 15068789

[B56] Miroshnikova Y. A. Nava M. M. Wickstrom S. A. (2017). Emerging roles of mechanical forces in chromatin regulation. J. Cell Sci. 130, 2243–2250. 10.1242/jcs.202192 28646093

[B57] Morita Y. Sato T. Higashiura K. Hirano Y. Matsubara F. Oshima K.. (2019). The optimal mechanical condition in stem cell-to-tenocyte differentiation determined with the homogeneous strain distributions and the cellular orientation control. Biol. Open 8:bio039164. 10.1242/bio.039164 31118166 PMC6550065

[B58] Na S. Trache A. Trzeciakowski J. Sun Z. Meininger G. A. Humphrey J. D. (2008). Time-dependent changes in smooth muscle cell stiffness and focal adhesion area in response to cyclic equibiaxial stretch. Ann. Biomed. Eng. 36, 369–380. 10.1007/s10439-008-9438-7 18214679

[B59] Nam H. Y. Pingguan-Murphy B. Amir Abbas A. Mahmood Merican A. Kamarul T. (2015). The proliferation and tenogenic differentiation potential of bone marrow-derived mesenchymal stromal cell are influenced by specific uniaxial cyclic tensile loading conditions. Biomech. Model Mechanobiol. 14, 649–663. 10.1007/s10237-014-0628-y 25351891

[B60] Nathan A. S. Baker B. M. Nerurkar N. L. Mauck R. L. (2011). Mechano-topographic modulation of stem cell nuclear shape on nanofibrous scaffolds. Acta Biomater 7, 57–66. 10.1016/j.actbio.2010.08.007 20709198 PMC2967658

[B61] Nuttall M. E. Gimble J. M. (2004). Controlling the balance between osteoblastogenesis and adipogenesis and the consequent therapeutic implications. Curr. Opin. Pharmacol.4, 290–294. 10.1016/j.coph.2004.03.002 15140422

[B62] Oh S. Brammer K. S. Li Y. S. J. Teng D. Engler A. J. Chien S.. (2009). Stem cell fate dictated solely by altered nanotube dimension. Proc. Natl. Acad. Sci. U.S.A. 106, 2130–2135. 10.1073/pnas.0813200106 19179282 PMC2650120

[B63] Park J. S. Chu J. S. Cheng C. Chen F. Chen D. Li S. (2004). Differential effects of equiaxial and uniaxial strain on mesenchymal stem cells. Biotechnol. Bioeng. 88, 359–368. 10.1002/bit.20250 15486942

[B64] Paul N. E. Denecke B. Kim B. S. Dreser A. Bernhagen J. Pallua N. (2017). The effect of mechanical stress on the proliferation, adipogenic differentiation and gene expression of human adipose-derived stem cells. J. Tissue Eng. Regen. Med. 12, 276–284. 10.1002/term.2411 28095649

[B65] Pek Y. S. Wan A. C. Ying J. Y. (2010). The effect of matrix stiffness on mesenchymal stem cell differentiation in a 3d thixotropic gel. Biomaterials 31, 385–391. 10.1016/j.biomaterials.2009.09.057 19811817

[B66] Pittenger M. F. Mackay A. M. Beck S. C. Jaiswal R. K. Douglas R. Mosca J. D.. (1999). Multilineage potential of adult human mesenchymal stem cells. Science 284, 143–147. 10.1126/science.284.5411.143 10102814

[B67] Qi M. C. Hu J. Zou S. J. Chen H. Q. Zhou H. X. Han L. C. (2008). Mechanical strain induces osteogenic differentiation: Cbfa1 and ets-1 expression in stretched rat mesenchymal stem cells. Int. J. Oral Maxillofac Surg. 37, 453–458. 10.1016/j.ijom.2007.12.008 18272346

[B68] Ramdas N. M. Shivashankar G. V. (2015). Cytoskeletal control of nuclear morphology and chromatin organization. J. Mol. Biol. 427, 695–706. 10.1016/j.jmb.2014.09.008 25281900

[B69] Roca-Cusachs P. Alcaraz J. Sunyer R. Samitier J. Farre R. Navajas D. (2008). Micropatterning of single endothelial cell shape reveals a tight coupling between nuclear volume in g1 and proliferation. Biophys. J. 94, 4984–4995. 10.1529/biophysj.107.116863 18326659 PMC2397343

[B70] Shivashankar G. V. (2011). Mechanosignaling to the cell nucleus and gene regulation. Annu. Rev. Biophys. 40:361–378. 10.1146/annurev-biophys-042910-155319 21391812

[B71] Song Y. Soto J. Chen B. Yang L. Li S. (2020). Cell engineering: biophysical regulation of the nucleus. Biomaterials 234, 119743. 10.1016/j.biomaterials.2019.119743 31962231

[B72] Tajik A. Zhang Y. Wei F. Sun J. Jia Q. Zhou W.. (2016). Transcription upregulation via force-induced direct stretching of chromatin. Nat. Mater. 15, 1287–1296. 10.1038/nmat4729 27548707 PMC5121013

[B73] Thakar R. G. Cheng Q. Patel S. Chu J. Nasir M. Liepmann D.. (2009). Cell-shape regulation of smooth muscle cell proliferation. Biophys. J. 96, 3423–3432. 10.1016/j.bpj.2008.11.074 19383485 PMC2718294

[B74] Thomas C. H. Collier J. H. Sfeir C. S. Healy K. E. (2002). Engineering gene expression and protein synthesis by modulation of nuclear shape. Proc. Natl. Acad. Sci. U.S.A. 99, 1972–1977. 10.1073/pnas.032668799 11842191 PMC122304

[B75] Torsoni A. S. Marin T. M. Velloso L. A. Franchini K. G. (2005). Rhoa/rock signaling is critical to fak activation by cyclic stretch in cardiac myocytes. Am. J. Physiol. Heart Circ. Physiol. 289, H1488–H1496. 10.1152/ajpheart.00692.2004 15923313

[B76] Trappmann B. Gautrot J. E. Connelly J. T. Strange D. G. Li Y. Oyen M. L.. (2012). Extracellular-matrix tethering regulates stem-cell fate. Nat. Mater. 11, 642–649. 10.1038/nmat3339 22635042

[B77] Uchibori R. Tsukahara T. Ohmine K. Ozawa K. (2014). Cancer gene therapy using mesenchymal stem cells. Int. J. Hematol. 99, 377–382. 10.1007/s12185-014-1537-7 24578184

[B78] Uhler C. Shivashankar G. V. (2017). Regulation of genome organization and gene expression by nuclear mechanotransduction. Nat. Rev. Mol. Cell Biol. 18, 717–727. 10.1038/nrm.2017.101 29044247

[B79] Versaevel M. Grevesse T. Gabriele S. (2012). Spatial coordination between cell and nuclear shape within micropatterned endothelial cells. Nat. Commun. 3:671. 10.1038/ncomms1668 22334074

[B80] Wang N. Tytell J. D. Ingber D. E. (2009). Mechanotransduction at a distance: mechanically coupling the extracellular matrix with the nucleus. Nat. Rev. Mol. Cell Biol. 10, 75–82. 10.1038/nrm2594 19197334

[B81] Wang X. Liu H. Zhu M. Cao C. Xu Z. Tsatskis Y.. (2018). Mechanical stability of the cell nucleus-roles played by the cytoskeleton in nuclear deformation and strain recovery. J. Cell Sci. 131:jcs209627. 10.1242/jcs.209627 29777038

[B82] Wang Y. K. Yu X. Cohen D. M. Wozniak M. A. Yang M. T. Gao L.. (2012). Bone morphogenetic protein-2-induced signaling and osteogenesis is regulated by cell shape, rhoa/rock, and cytoskeletal tension. Stem. Cells Dev. 21, 1176–1186. 10.1089/scd.2011.0293 21967638 PMC3328763

[B83] Wu M. Chen G. Li Y. P. (2016). Tgf-beta and bmp signaling in osteoblast, skeletal development, and bone formation, homeostasis and disease. Bone Res. 4:16009. 10.1038/boneres.2016.9 27563484 PMC4985055

[B84] Xu B. Song G. Ju Y. Li X. Song Y. Watanabe S. (2012). Rhoa/rock, cytoskeletal dynamics, and focal adhesion kinase are required for mechanical stretch-induced tenogenic differentiation of human mesenchymal stem cells. J. Cell Physiol. 227, 2722–2729. 10.1002/jcp.23016 21898412

[B85] Yorukoglu A. C. Kiter A. E. Akkaya S. Satiroglu-Tufan N. L. Tufan A. C. (2017). A concise review on the use of mesenchymal stem cells in cell sheet-based tissue engineering with special emphasis on bone tissue regeneration. Stem. Cells Int. 2017:2374161. 10.1155/2017/2374161 29230248 PMC5694585

[B86] Zhao B. Ye X. Yu J. Li L. Li W. Li S.. (2008). Tead mediates yap-dependent gene induction and growth control. Genes Dev. 22, 1962–1971. 10.1101/gad.1664408 18579750 PMC2492741

[B87] Zwolanek D. Satue M. Proell V. Godoy J. R. Odorfer K. I. Flicker M.. (2017). Tracking mesenchymal stem cell contributions to regeneration in an immunocompetent cartilage regeneration model. JCI Insight 2:e87322. 10.1172/jci.insight.87322 29046476 PMC5846895

